# Retraction Note: Conductive Gels: Properties and Applications of Nanoelectronics

**DOI:** 10.1186/s11671-022-03746-9

**Published:** 2022-11-14

**Authors:** Nguyen Dinh Trung, Dinh Tran Ngoc Huy, Maria Jade Catalan Opulencia, Holya A. Lafta, Azher M. Abed, Dmitry Olegovich Bokov, Kahramon Shomurodov, Hoang Van Thuc Master, Ali Thaeer Hammid, Ehsan Kianfar

**Affiliations:** 1 National Economics University (NEU), Hanoi, Vietnam; 2 Banking University HCMC, Ho Chi Minh City, Vietnam; 3 International University of Japan, Niigata, Japan; 4 College of Business Administration, Ajman University, Ajman, United Arab Emirates; 5 Al-Nisour University College, Baghdad, Iraq; 6 Department of Air Conditioning and Refrigeration, Al-Mustaqbal University College, Babylon, Iraq; 7 Institute of Pharmacy, Sechenov First Moscow State Medical University, 8 Trubetskaya St., Bldg. 2, Moscow, Russian Federation 119991; 8 Laboratory of Food Chemistry, Federal Research Center of Nutrition, Biotechnology and Food Safety, 2/14 Ustyinsky Pr, Moscow, Russian Federation 109240; 9 Department of Maxillo-Facial Surgery, Tashkent State Dental Institute, Makhtumkuli 103, Tashkent, Uzbekistan 100147; 10 Thai Nguyen University, University of Information and Communication Technology, Thái Nguyên, Vietnam; 11 Computer Engineering Department, Imam Ja’afar Al-Sadiq University, Baghdad, Iraq; 12 Department of Chemical Engineering, Arak Branch, Islamic Azad University, Arak, Iran; 13 Young Researchers and Elite Club, Gachsaran Branch, Islamic Azad University, Gachsaran, Iran

**Retraction Note: Nanoscale Research Letters (2022) 17:50** 10.1186/s11671-022-03687-3

The Editors-in-Chief have retracted this article because an investigation by the Publisher has shown evidence of authorship manipulation. The contributions of the listed authors cannot be confirmed and the Editors-in-Chief no longer have confidence in the content of the article. Ehsan Kianfar disagrees with this retraction. Nguyen Dinh Trung, Dinh Tran Ngoc Huy, Maria Jade Catalan Opulencia, Holya A. Lafta, Azher M. Abed, Hoang Van Thuc Master and Ali Thaeer Hammid have not responded to correspondence from the Publisher about this retraction. The Publisher was not able to find a current email address for Dmitry Olegovich Bokov and Kahramon Shomurodov.

